# Trends in quality of care among patients with incident heart failure in Denmark 2003–2010: a nationwide cohort study

**DOI:** 10.1186/1472-6963-13-391

**Published:** 2013-10-05

**Authors:** Anne Nakano, Søren Paaske Johnsen, Birgitte Lidegaard Frederiksen, Marie Louise Svendsen, Carsten Agger, Inge Schjødt, Kenneth Egstrup

**Affiliations:** 1 Department of Clinical Epidemiology, Aarhus University Hospital, Aarhus, Denmark; 2 The Danish Clinical Registers, Audit Unit West, Olof Palmes Allé 15, Aarhus, Denmark; 3 Danish Health and Medicines Authority, Copenhagen, Denmark; 4 Research Centre for Prevention and Health, Glostrup, Denmark; 5 Department of Cardiology, Aarhus University Hospital, Aarhus, Denmark; 6 Department of Cardiology, Odense University Hospital, Svendborg Hospital, Svendborg, Denmark

**Keywords:** Quality indicators, Registries, Treatment and care, Heart failure

## Abstract

**Background:**

The treatment of heart failure (HF) is complex and the prognosis remains serious. A range of strategies is used across health care systems to improve the quality of care for HF patients. We present results from a nationwide multidisciplinary initiative to monitor and improve the quality of care and clinical outcome of HF patients using indicator monitoring combined with systematic auditing.

**Methods:**

We conducted a nationwide, population-based prospective study using data from the Danish Heart Failure Registry. The registry systematically monitors and audits the use of guideline recommended processes of care at Danish hospital departments treating incident HF patients. We identified patients registered between 2003 and 2010 (n = 24504) and examined changes in use of recommended processes of care and 1-year mortality.

**Results:**

The use of the majority of the recommended processes of care increased substantially from 2003 to 2010: echocardiography (from 62.7% to 90.5%; Relative Risk (RR) 1.45 (95% CI, 1.39-1.50)), New York Heart Association classification (from 29.4% to 85.5%; RR 2.91 (95% CI, 2.69-3.14)), betablockers (from 72.6% to 88.3%; RR 1.23 (95% CI, 1.15-1.29)), physical training (from 5.6% to 22.8%; RR 4.04 (95% CI, 2.96-4.52)), and patient education (from 49.3% to 81.4%; RR 1.65 (95% CI, 1.52-1.80)). Use of ACE/ATII inhibitors remained stable (from 92.0% to 93.2%; RR 1.01 (95% CI, 0.99-1.04)). During the same period, 1-year mortality dropped from 20.5% to 12.8% (adjusted Hazard Ratio 0.79 (95% CI, 0.65-0.96).

**Conclusions:**

Use of guideline recommended processes of care has improved among patients with incident HF included in the Danish Heart Failure Registry between 2003 and 2010. During the same period, a decrease in mortality was observed.

## Background

Heart failure (HF) is an important cause of morbidity and mortality worldwide [1]. The prevalence of HF is increasing globally due to ageing populations in the developed countries, improved survival in patients suffering from coronary events and the success achieved in postponing coronary events using effective preventive measures [2-8]. HF care has developed substantially in recent decades and clinical trials have established several new therapies which have improved clinical outcomes for patients with HF and reduced left ventricular ejection fraction (LVEF) [8]. However, treatment guidelines are adopted slowly and applied inconsistently and may thus not result in the expected improvements in patient care and clinical outcomes [9-12]. Consequently, in many health care systems, major efforts are made to implement recommended guidelines [13]. However, population-based data on the implementation of the recommendations in everyday clinical practice and the possible impact on patient outcomes are still sparse [14].

In Denmark, the quality of care for patients with incident HF has been monitored and audited continuously in a national multidisciplinary quality improvement program since 2003. We aimed to examine whether the quality of care and the mortality among incident HF patients hospitalized in Denmark has changed following the introduction of the program.

## Methods

### The Danish heart failure registry

All Danish residents (approximately 5.5 million) have free access to hospital care provided by the tax-financed Danish National Health Service. The Danish Heart Failure Registry (DHFR) was established as a part of a larger nationwide initiative, The Danish National Indicator Project, in 2003 in order to monitor and improve the quality of care for HF patients [15].

Since 2003, the DHFR has monitored and supported implementation of evidence-based treatment and care for incident hospitalized HF patients. Participation is mandatory for all hospital units and outpatient cardiology clinics treating patients with HF. However, not all hospitals were able to report to the register when it was launched in 2003.

The prognostic factors recorded in the registry as well as the evidence-based quality of care indicators were identified by a multidisciplinary national expert panel based on national [15] and international guidelines from the American College of Cardiology, the American Heart Association [16], and the European Society of Cardiology [17], supplemented by a systematic literature review. The expert panel followed a structured, rigorous and evidence-based guideline-driven process to develop pathways and tools for clinicians in hospitals and outpatient HF clinics in order to ensure data accuracy by standardizing procedures. This included developing detailed instructions for the data collection with strict data definitions ensuring that clinicians register data in the same manner at every hospital, as well as providing regular performance reports to the participating hospitals as also done in the American Heart Association Get With the Guidelines Program for Heart Failure [18]. The feasibility of collecting the required data in routine clinical settings, and the ability of the processes to reflect the multidisciplinary efforts involved in modern HF care, were also considered.

The expert panel identified 6 process indicators and 1 outcome indicator (Table 1), and a number of prognostic factors (Table 2).

**Table 1:** Processes of care monitored in the Danish heart failure registry

**Processes of care**	
Echocardiography	Proportion of patients who undergo echocardiography
NYHA classification	Proportion of patients who undergo NYHA classification
Medication (ACE/ATII inhibitors)	Proportion of patients with reduced systolic function (LVEF below 40%) who is treated with ACE/ATII inhibitors
Medication (Betablockers)	Proportion of patients with reduced systolic function (LVEF below 40%) who is treated with betablockers
Physical training	Proportion of patients with reduced systolic function (LVEF below 40%) referred to individual physical training
Patient education	Proportion of patients with reduced systolic function (LVEF below 40%) who started a structured patient education (inclusive nutrition, physical training, understanding medical treatment, risk factors and symptoms of the disease)
1-year mortality	Proportion of patients who die within one year of admission to a hospital or first outpatient contact

*NYHA* New York Heart Association, *ACEI*/*ATII* Angiotensin Converting Enzyme/Angiotensin II Antagonist inhibitors, *LVEF* Left Ventricular Ejection Fraction.

**Table 2:** **Baseline characteristics among patients diagnosed with incident heart failure in Denmark between 2003 and 2010** (**N** = **24504**)

	**N (%)**
Total	24504 (100)
**Age** mean (SD)	70.8 (13.2)
**Gender**	
Male	15607 (63.7)
Female	8897 (36.3)
**Left Ventricular Ejection Fraction (LVEF)**	
LVEF < 25	6609 (27.0)
25 ≤ LVEF ≤ 35	7803 (31.8)
35 < LVEF ≤ 40	3498 (14.3)
40 < LVEF < 50	2287 (9.3)
LVEF ≥ 50	1134 (4.6)
Missing	3173 (13.6)
**New York Heart Association (NYHA) classification**	
NYHA-class 1	1912 (7.8)
NYHA-class 2	8209 (33.5)
NYHA-class 3	4462 (18.8)
NYHA-class 4	459 (1.9)
Missing	9462 (38.6)
**Previous Acute Myocardial Infarction (AMI)**	
Yes	8046 (32.8)
No	14859 (60.6)
Missing	1599 (6.5)
**Stroke**	
Yes	2561 (10.5)
No	19576 (79.9)
Missing	2367 (9.7)
**Chronic Obstructive Pulmonary Disease (COPD)**	
Yes	3759 (15.3)
No	18480 (75.4)
Missing	2265 (9.2)
**In treatment for hypertension**	
Yes	8335 (34.0)
No	14378 (58.7)
Missing	1791 (7.3)
**Diabetes**	
Yes	4530 (18.5)
No	18362 (74.9)
Missing	1612 (6.6)
**Alcohol intake**	
Maximum 14 drinks for women and 21 for men per week	16683 (68.1)
More than 14 drinks for women and 21 for men per week	1639 (6.7)
Missing	6010 (25.7)
**Smoking habits**	
Smoker	7101 (29.0)
Non-smoker	17335 (70.8)
Missing	48 (0.2)

*SD* Standard Deviation, *LVEF* Left Ventricular Ejection Fraction, *NYHA* New York Heart Association, *AMI* Acute Myocardial Infarction, *COPD* Chronic Obstructive Pulmonary Disease.

It was not possible to differentiate between inpatients and outpatients until 2006. Results from 2006 to 2010 are available in the supplementary online material.

Data are registered for HF patients admitted to hospital or at the first outpatient visit as part of the clinical routine by cardiologists and nursing staff.

The use of 2 processes of care (echocardiography and New York Heart Association classification (NYHA classification) and 1-year mortality is monitored in all patients. The remaining processes of care (Angiotensin Converting Enzyme/Angiotensin II antagonist (ACE/ATII) inhibitors, betablockers, physical training, and patient education) are only monitored in patients with systolic HF (LVEF ≤ 40%).

Regular, structured audits are conducted on a national, regional, and local basis, and include validation of the completeness of patient registration against local hospital discharge registries and the National Registry of Patients [19]. Furthermore, every 3 months, the participating departments receive feedback data on their performance regarding the process indicators as well as unadjusted data on mortality. The feedback data are reported on a web-based information system allowing each participating hospital to review its performance data, and benchmark them against the region and the whole country.

### Study population

The study population included patients with a first time hospitalization (including in- and out-patients) with HF as the primary diagnosis. Diagnoses are made by an experienced cardiologist, using the ESC guidelines for definition of HF, and recorded according to the International Classification of Diseases, 10^th^ revision (ICD-10) (Codes: I11.0, I13.0, I13.2, I42.0, I42.6, I42.7, I42.8, I42.9, I50.0, I50.1, I50.2, I50.3, I50.8, I50.9).

Outpatients had typically previously been admitted to a cardiology ward with acute myocardial infarction and had during the admission developed symptoms of HF. After treatment for the acute myocardial infarction, the patients were then referred to an outpatient cardiology clinic for treatment of the HF.

The decision of recording a patient in the registry is always made by a senior cardiologist to ensure the validity of the HF diagnosis [7,20]. Each patient was only included once in the analyses. Patients were 18 years of age or older and Danish residents. They were enrolled irrespective of their left ventricular function. The total number of patients registered in the DHFR was 24510 in the study period, but six patients were under 18 years of age, and therefore excluded, leaving 24504 patients for analysis.

A total of 41 hospitals and 54 departments were represented in this study. The hospitals and departments, which represent all hospitals and departments responsible for treating HF patients in Denmark, were identified by Danish Regions, which are responsible for running the hospitals. For the majority of the departments, the completeness of the registration of patients was 98-100% in 2010 compared with local hospital discharge registries and the Danish National Registry of Patients [16].

### Data on patient characteristics and mortality

Data on patient characteristics, including gender, age, comorbidity, left ventricular ejection fraction and NYHA classification as well as alcohol intake and smoking habits, were obtained from the DHFR. Information on vital status (1-year mortality) was obtained from the Danish Civil Registration System [21], which maintains electronic records of changes in the vital status of all residents. Each record carries a unique 10-digit civil registration number, which is used in all Danish population based registries and enables unambiguous linkage among these registries. The study was approved by the Danish Data Protection Agency (J.no. 2008-41-2072), the DHFR, and the Danish Ministry of Health.

### Statistics

We computed the proportion of patients receiving the individual processes of care among those eligible as well as the proportion of HF patients who died within 1 year of admission or first contact, both overall for the entire study period and according to calendar year (2003–2010). Comparisons over time were made using binary regression to compute the relative risk (RR) with 95% confidence intervals (CI) using 2003 as reference. A composite quality of care measure was also computed for each department. This measure was defined as the total number of received processes of care divided by the total number of processes of care relevant to the patients admitted to the individual department.

Analyses on mortality were conducted for the entire study population and stratified according to LVEF (40% or less vs. more than 40%). For some patients, data on one or more of the covariates were missing (Table 2). We used multiple imputation to impute the missing values assuming that data was missing at random (stata command: ice) [22-24]. We created 5 datasets based on the following covariates: age, gender, left ventricular ejection fraction, previous acute myocardial infarction, stroke, chronic obstructive pulmonary disease, diabetes, alcohol intake, smoking habits and patients in treatment for hypertension. The proportion of patients, for whom data on these variables were missing, varied between 0.0%-25.7%.

We compared 1-year mortality between patients from 2010 and 2003, respectively using multivariable Cox proportional hazards regression, while controlling for the patient characteristics presented in Table 2 (except for NYHA class, due to a high proportion missing data and inpatient/outpatient status, which was not registered before 2006).

Data were analysed using Stata 10.0 (StataCorp LP, College Station, Texas).

## Results

### Processes of care

Baseline characteristics of the total patient population are presented in Table 2. In the Additional file 1: Table S1, the characteristics are listed according to year of registration (2003 to 2010). The proportion of patients receiving the individual processes of care increased substantially between 2003 and 2010 (Table 3), i.e., use of echocardiography (RR 1.45, 95% CI, 1.39-1.50), NYHA classification (RR 2.91, 95% CI, 2.69-3.14) , betablockers (RR 1.23, 95% CI 1.15-1.29), physical training (RR 4.04, 95% CI, 2.96-5.52) and patient education (RR 1.65, 95% CI, 1.52-1.80). The only exception was the use of ACE/ATII inhibitors, where no overall changes were observed (RR 1.01, 95% CI, 0.99-1.04).

**Table 3:** **Received processes of care among patients diagnosed with incident heart failure in Denmark between 2003 and 2010** (**N** = **24504**)

**Total**	**Year 2003 to 2010**	**Year 2003**	**Year 2010**	**Crude RR (95% CI)**
	**N (%)**	**N (%)**	**N (%)**	
	**24504 (100)**	**1624 (100)**	**3809 (100)**	
**Processes of care**				
Echocardiograph performed	19419 (79.5)	1010 (62.7)	3430 (90.5)	1.45 (1.39-1.50)
NYHA classification assessed	15042 (61.6)	475 (29.4)	3237 (85.5)	2.91 (2.69-3.14)
ACE/ATII inhibitors given	12565 (93.0)	446 (92.0)	2628 (93.2)	1.01 (0.99-1.04)
Betablockers given	11272 (84.4)	350 (72.6)	2489 (88.3)	1.23 (1.15-1.29)
Physical training	2278 (15.9)	39 (5.6)	631 (22.8)	4.04 (2.96-5.52)
Patient education	9852 (70.0)	273 (49.3)	2281 (81.4)	1.65 (1.52-1.80)

*NYHA* New York Heart Association, *ACE*/*ATII* Angiotensin Converting Enzyme/Angiotensin II Antagonist inhibitors.

**Figure 1:**
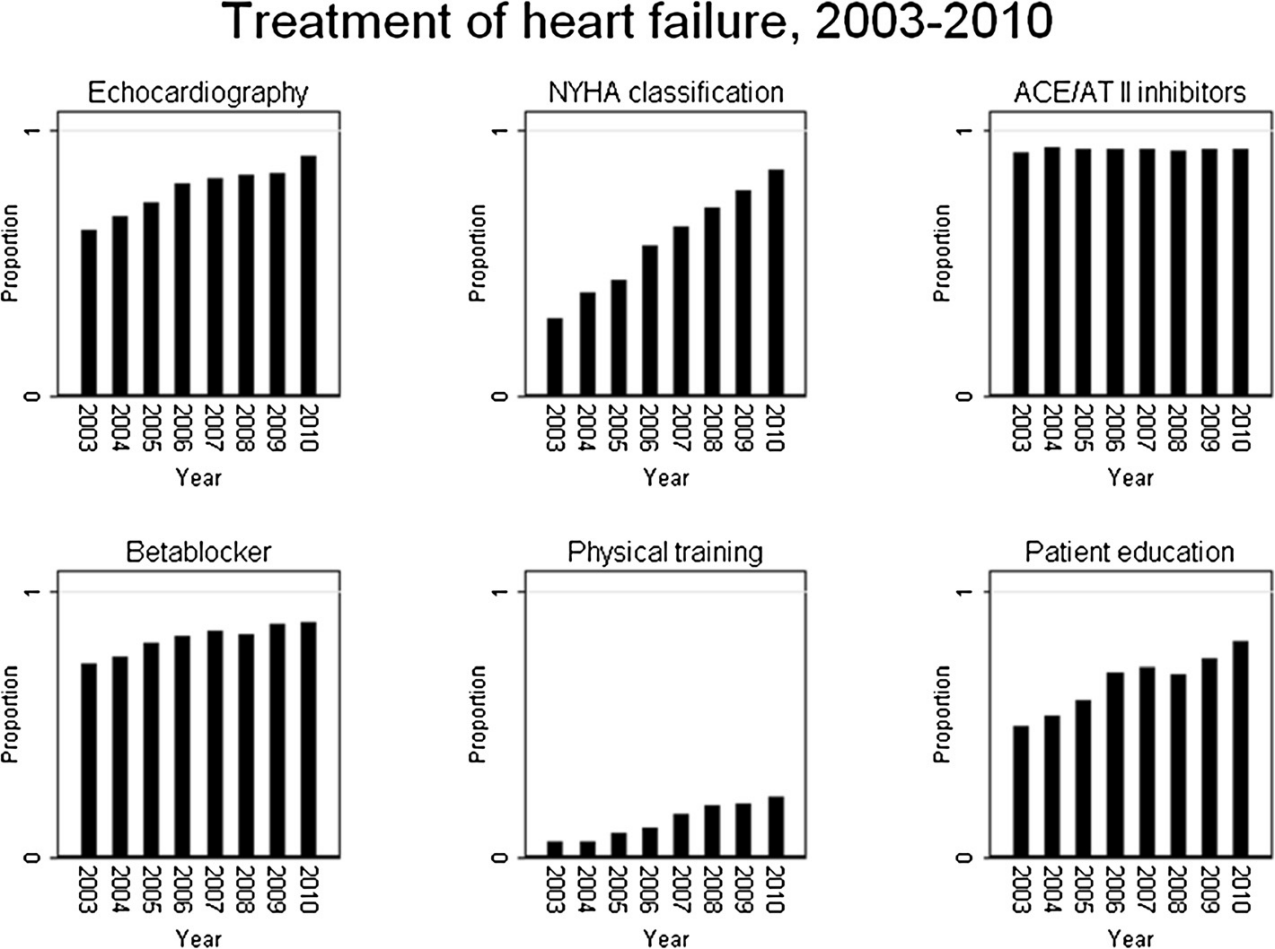
**Proportion of patients receiving the recommended processes of care among patients diagnosed with incident heart failure in Denmark 2003**–**2010.** NYHA classification: New York Heart Association classification, ACE/ATII inhibitors: Angiotensin Converting Enzyme/ Angiotensin II antagonist inhibitor.

Figure 1 shows the increase in the proportion of patients who received the recommended processes of care between 2003 and 2010.

**Figure 2:**
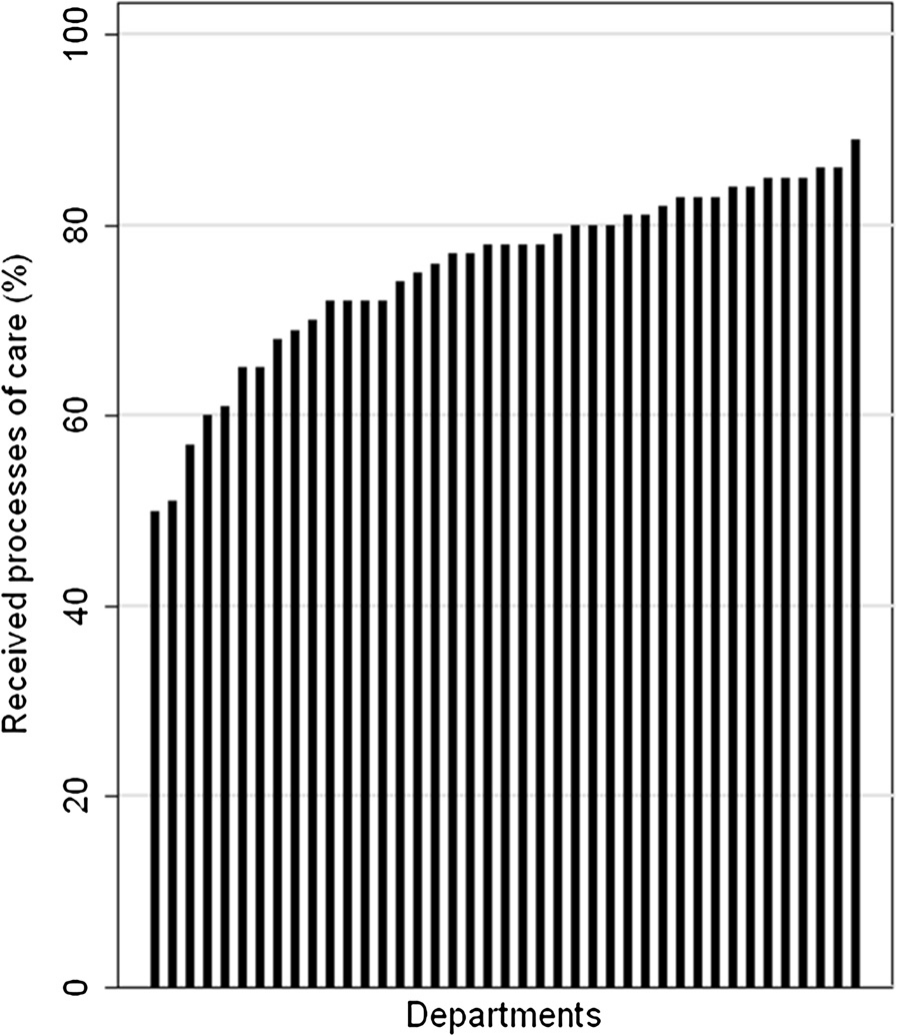
**Variation between Danish hospital departments in overall proportion of delivered processes of recommended care to patients with incident heart failure in 2010.** The bars represent individual hospital departments.

Figure 2 presents the overall composite process indicator, reflecting the proportion of all recommended processes of care that was delivered in 2010 at the individual departments. Although overall improvements were observed for most processes of care, substantial variation in quality of care remains among hospital departments treating patients with incident HF in Denmark. The proportion of delivered recommended processes of care varied between 50% and 89% across the departments.

### Mortality

Overall 1-year all-cause mortality among patients registered in the DHFR decreased from 20.5% in 2003 to 12.8% in 2010 (Table 4).

**Table 4:** **One**-**year mortality among patients diagnosed with incident heart failure in Denmark in 2010 vs. 2003**

	**Mortality 2003**	**Mortality 2010**	**Unadjusted HR**	**Adjusted HR** *	**Adjusted HR †**
	**N/total (%)**	**N/total (%)**	**(95% CI)**	**(95% CI)**	**(95% CI)**
Total	333/1624 (20.5)	488/3809 (12.8)	0.59 (0.51-0.67)	0.65 (0.56-0.75)	0.79 (0.65-0.96)
LVEF ≤40%	277/1379 (20.1)	408/3141 (13.0)	0.61 (0.52-0.71)	0.67 (0.57-0.78)	0.85 (0.69-1.05)
LVEF >40%	56/245 (22.9)	79/668 (11.8)	0.47 (0.31-0.74)	0.58 (0.40-0.84)	0.51 (0.30-0.89)

*Hazard Ratio (HR) adjusted for age and gender.

† Hazard Ratio (HR) adjusted for the following patient characteristics: age, gender, LVEF, previous acute myocardial infarction, stroke, chronic obstructive pulmonary disease, diabetes, alcohol intake, smoking habits, and in treatment for hypertension.

*LVEF* Left Ventricular Ejection Fraction.

Overall and stratified according to selected patient characteristics.

The overall adjusted hazard ratio (HR) for 1-year mortality was 0.79 (95% CI, 0.65-0.96) after multivariable adjustment for patient characteristics (age, gender, LVEF, previous acute myocardial infarction, stroke, chronic obstructive pulmonary disease, diabetes, alcohol intake, smoking habits and in treatment for hypertension), when comparing patients diagnosed in 2010 with patients diagnosed in 2003. Analyses were also stratified for LVEF (Table 4). The improvements in mortality appeared to be better in patients with preserved ejection fraction compared to patients with reduced ejection fraction. The confidence intervals were, however, overlapping.

## Discussion

We found that implementation of indicator monitoring for HF care in Denmark has been associated with substantial improvements in the use of guideline recommended processes of care among patients registered in the national HF registry. Similar results have been observed in at least two other major quality improvement initiatives: The Registry to Improve the Use of Evidence-Based Heart Failure Therapies in the Outpatient Setting (IMPROVE-HF) for outpatient cardiology practices where 7 quality measures were assessed and significant improvement achieved for 5 of the measures. Identical to our study, they did not reach statistical significance in angiotensin-converting enzyme inhibitor/angiotensin receptor blocker [10]. Likewise, The Get With the Guidelines Programme for Heart Failure demonstrated better processes of care as well as improved performance over time in hospitals following the guidelines compared to hospitals that did not [18], as also shown in our study.

Furthermore, we observed a reduced 1-year mortality rate among Danish HF patients included in the DHFR when comparing patients diagnosed in 2010 with patients diagnosed in 2003.

Direct comparisons with other studies is somewhat hampered by the use of different study designs (population-based vs. selected institutions) and patient populations (prevalent vs. incident patients, inpatients vs. outpatients). However, the baseline profile of our patients appears to be comparable with the profile reported in a number of other studies [10,11,25,26]. Furthermore, our findings are in general in accordance with and extend findings from other existing studies, which have addressed the effects of implementation of clinical guidelines and indicator monitoring. According to two studies by Fonarow et al. based on data from OPTIMIZE-HF (The Organized Program to Initiate Lifesaving Treatment in Hospitalized Patients With Heart Failure) and IMPROVE HF (Primary results of the Registry to Improve the Use of Evidence-Based Heart Failure Therapies in the Outpatient Setting cohort), use of guideline recommended therapies, including discharge instructions, assessment of left ventricular function, ACE inhibitors or angiotensin II receptor blockers (ARBs) and betablockers at discharge, was associated with lower mortality [10,27]. There are conflicting results, though, as Heidenreich et al. found a decrease in the 30-day readmission rate, but not in the 30-day mortality rate using data from the American Heart Association’s Get With The Guidelines Program. The inconsistency may be related to the studied processes of care and outcomes. The Get With The Guidelines Program focused on documentation of LVEF, use of ACE inhibitors if LVEF was less than 40%, as well as discharge instructions and smoking cessation. A stronger association between the processes of care and short-term mortality could possibly have been found if the use of betablockers or aldosterone antagonists had also been assessed since use of these drugs has been shown to improve survival in randomized trials [18].

Our population included both patients with and without preserved ejection fraction, although it should be noted that the proportion of included patients with preserved ejection fraction was quite small (4.6%). Although we found no statistically significant difference in the improvements in mortality during the study period for patients with versus patients without preserved ejection fraction, we did observe an indication of a stronger improvement among patients with preserved ejection fraction. This is noteworthy as the existing evidence base for treatment of patients with preserved ejection fraction is weak as no treatment has yet been shown to reduce morbidity and mortality in this patient group [17].

Studies on other cardiovascular patient groups, including patients with acute coronary syndrome and stroke, have also provided evidence for the effectiveness of optimizing guideline recommended care among patients encountered in real-world clinical practice [28-31]. Our study appear to add further support to the important role of clinical guidelines and HF programmes as tools for bridging the gap between research and routine clinical practice.

In the DHFR, the continuous monitoring of the quality of care is supplemented by regular audits and public reporting and release of the performance data from the individual departments. Such steps may further ensure commitment and active involvement of the stakeholders, including clinicians, administrators, patients and politicians. However, challenges remain as demonstrated by the substantial variation between the hospital departments in the overall quality of care even after years of monitoring and auditing.

### Strengths and limitations

The strengths of our study include the prospective nationwide population-based design and the large number of patients included, as well as the fact that registration is mandatory to all hospitals in Denmark treating patients with HF, keeping in mind that not all hospitals were capable of beginning registration at the same time. In addition, thorough efforts are made to ensure data validity in the DNIP. Regular multidisciplinary structured audits are conducted, which include validation of the completeness of patient registration against hospital discharge registries and discussion and exchange of experience and knowledge in order to explain the results and implement improvements [15].

The main limitation is the observational nature of our study, which precludes firm conclusions about causality, in particular with regards to the findings on mortality. The completeness of the registration of a patient is important in this context and it should therefore be noted that the number of patients included in DHFR per year clearly increased during the study period (from 1624 patients in 2003 to 3809 patients in 2010). This reflected an increasing completeness of the DHFR as all relevant hospitals and departments began reporting to the registry at some point between 2003 and 2010. The DHFR aims to include all incident patients admitted with HF as the primary diagnosis. Consequently, the DHFR will not reflect the incidence of HF in the general Danish population. The low proportion of patients with preserved ejection fraction (4.6%) also indicates that not all hospitalized HF patients were included since it has been estimated that as many as 20% to 60% of HF patients have a normal or near normal LVEF [8]. However, the high completeness of the DHFR compared with hospital discharge registries, indicates that the registry probably did cover the vast majority of incident HF patients admitted to Danish hospitals with HF as the primary diagnosis during the study period.

Other factors, besides the nationwide initiative, may potentially have contributed to the improved quality of care and lower mortality including a major structural reform of the Danish health care system in 2007 and a generally increased awareness among clinicians of guideline recommendations and in particular increased focus on caring for persons with chronic conditions. The latter has during the study period been specifically stimulated by reports from the National Board of Health presenting different options for improving care for those with chronic conditions as well as the publication of disease management programs for persons with chronic conditions [32,33]. Changes over time in the prognostic profile of the patients with incident HF, e.g., the increase in the proportion of patients being treated as outpatients, is another important issue. Although we controlled for a range of well-established prognostic factors in the analyses on changes in mortality over time, data was not available on all relevant factors (e.g., creatinine levels and use of implantable cardioverter defibrillators, cardiac resynchronization therapy, and aldosterone antagonist medications). In addition, the proportion of patients for whom data were missing was substantial for some of the registered variables, e.g., NYHA class (38.6%). Assuming that our data were missing at random, we used multiple imputation to account for missing data on the covariates included in the multivariable analyses on mortality. This approach is not without pitfalls, in particular due to the difficulties with assessing whether data are truly missing at random. However, the implications of using the technique appeared modest in the analyses, as all analyses indicated a lower mortality among patients diagnosed in 2010 patients compared with patients diagnosed in 2003 patients independently on how the available covariates were included in the multivariable analyses (data not shown).

Finally, the inherent risk of gaming in top-down initiated quality improvement initiatives such as the DHFR should not be forgotten. “Gaming” is here understood as reactive subversion such as “hitting the target and missing the point” or reducing performance where targets do not apply. The phenomenon is described by the economist Charles Goodhart, who following the failure of the UK government’s reliance on money supply targets in the 1980s to control inflation, to stated: “Any observed statistical regularity will tend to collapse once pressure is placed on it for control purposes’ because actors will change their conduct when they know that the data they produce will be used to control them” (Goodhart [34], p. 96). However, the risk of gaming in the DHFR was probably quite low due to the regular national, regional and local multidisciplinary clinical audits, where data collection and performance was discussed in details. Furthermore, the data validity was also ensured by multiple audits of medical journals and consistently updated manuals with explicit instructions to the staff involved in data collection.

## Conclusions

In conclusion, use of guideline recommended processes of care has improved substantially between 2003 and 2010 following the initiation of systematic quality of care monitoring among incident HF patients admitted to Danish hospitals and registered in the DHFR. The 1-year mortality appear to have decreased during the same period.

## Competing interest

The authors declare that they have no competing interest.

## Authors’ contributions

AN, SPJ, MLS and KE designed the study. AN was the principal investigator and lead author in the analysis of the data and wrote the draft of the manuscript. All authors participated in the interpretation of the findings. All authors took part in reviewing and editing the manuscript and approved the final version to be published.

## Pre-publication history

The pre-publication history for this paper can be accessed here:

http://www.biomedcentral.com/1472-6963/13/391/prepub

## Supplementary Material

Additional file 1: Table S1 Baseline characteristics of 24504 incident heart failure patients in Denmark registered in the Danish National Indicator Project between 2003 and 2010, and each separate year from 2003 to 2010. Click here for file
