# Supplementary material for: Trends in quality of care among patients with incident heart failure in Denmark 2003–2010: a nationwide cohort study
Source: BMC Health Serv Res. 2013 Oct 5;13:391. doi: 10.1186/1472-6963-13-391 (PMC3851278; doi:10.1186/1472-6963-13-391)
Supplement: Additional file 1: Table S1 — Baseline characteristics of 24504 incident heart failure patients in Denmark registered in the Danish National Indicator Project between 2003 and 2010, and each separate year from 2003 to 2010. [file 1472-6963-13-391-S1.doc]

**Additional file 1: Table S1**

**Baseline characteristics of 24504 incident heart failure patients in Denmark registered in the Danish National Indicator Project between 2003 and** 2010, and each separate year from 2003 to 2010

|  | 2003-2010: N (%) | 2003: N (%) | 2004: N (%) | 2005: N (%) | 2006: N (%) | 2007: N (%) | 2008: N (%) | 2009: N (%) | 2010: N (%) |
| --- | --- | --- | --- | --- | --- | --- | --- | --- | --- |
| **Gender Total** | 24504 (100) | 1624 (100) | 2777 (100) | 2987 (100) | 3005 (100) | 3453 (100) | 3405 (100) | 3444 (100) | 3809 (100) |
| Total Male | 15607 (63.7) | 1009 (62.1) | 1657 (59.7) | 1849 (61.9) | 1894 (63.0) | 2223 (64.4) | 2210 (65.0) | 2267 (65.8) | 2498 (65.6) |
| Total Female | 8897 (36.3) | 615 (37.9) | 1120 (40.3) | 1138 (38.1) | 1111 (37.0) | 1230 (35.6) | 1195 (35.1) | 1177 (34.2) | 1311 (34.4) |
| **Age** |  |  |  |  |  |  |  |  |  |
| Age, mean (SD) | 70.8 (13.2) | 72.0 (13.0) | 71.9 (13.2) | 71.5 (13.4) | 71.1 (13.3) | 70.4 (13.1) | 70.3 (13.2) | 70.4 (13.1) | 70.0 (13.0) |
| Age, Male Mean (SD) | 68.7 (12.8) | 69.6 (12.3) | 69.5 (12.8) | 68.9 (13.3) | 68.6 (13.1) | 68.7 (12.6) | 68.5 (12.8) | 68.7 (12.9) | 68.5 (12.6) |
| Age, Female Mean (SD) | 74.2 (13.1) | 76.1 (13.0) | 74.4 (12.9) | 75.7 (12.6) | 75.3 (12.5) | 73.4 (13.5) | 73.6 (13.3) | 73.8 (12.9) | 72.7 (13.4) |
| **In/outpatients*** |  |  |  |  |  |  |  |  |  |
| Inpatients |  |  |  |  | 1885 (62.7) | 2074 (60.1) | 1980 (58.2) | 1905 (55.3) | 1840 (48.3) |
| Outpatients |  |  |  |  | 1104 (36.7) | 1373 (39.8) | 1425 (41.9) | 1539 (44.7) | 1967 (51.6) |
| Missing |  |  |  |  | 16 (0.5) | 6 (0.2) | 0 | 0 | 2 (0.05) |
| **LVEF** |  |  |  |  |  |  |  |  |  |
| LVEF < 25 | 6609 (27.0) | 452 (27.8) | 823 (29.6) | 959 (32.1) | 1088 (36.2) | 937 (27.1) | 714 (21.0) | 773 (22.4) | 863 (22.6) |
| 25 ≤ LVEF ≤ 35 | 7803 (31.8) | 331 (20.4) | 631 (22.7) | 736 (24.6) | 848 (28.2) | 1131 (32.8) | 1311 (38.5) | 1282 (37.2) | 1533 (40.2) |
| 35 < LVEF ≤ 40 | 3498 (14.3) | 187 (11.5) | 347 (12.5) | 405 (13.6) | 456 (15.2) | 502 (14.5) | 457 (13.4) | 522 (15.2) | 622 (16.3) |
| 40 < LVEF < 50 | 2287 (9.3) | 168 (10.3) | 313 (11.3) | 328 (11.0) | 257 (8.6) | 305 (8.8) | 285 (8.4) | 316 (9.2) | 315 (8.3) |
| LVEF ≥ 50 | 1134 (4.6) | 1 (0.06) | 3 (0.1) | 5 (0.2) | 10 (0.3) | 214 (6.2) | 320 (9.4) | 266 (7.7) | 315 (8.3) |
| Missing | 3173 (13.0) | 485 (29.9) | 660 (23.8) | 554 (18.6) | 346 (11.5) | 364 (10.5) | 318 (9.3) | 285 (8.3) | 161 (4.2) |
| **NYHA-classification** |  |  |  |  |  |  |  |  |  |
| NYHA-class 1 | 1912 (7.8) | 46 (2.9) | 121 (4.4) | 153 (5.1) | 206 (6.9) | 297 (8.6) | 294 (8.6) | 366 (10.6) | 429 (11.3) |
| NYHA-class 2 | 8209 (33.5) | 250 (15.4) | 567 (20.4) | 679 (22.7) | 892 (29.7) | 1130 (32.7) | 1314 (38.6) | 1470 (42.7) | 1907 (50.1) |
| NYHA-class 3 | 4462 (18.2) | 154 (9.5) | 343 (12.4) | 429 (14.4) | 550 (18.3) | 696 (20.2) | 718 (21.1) | 747 (21.7) | 825 (21.7) |
| NYHA-class 4 | 459 (1.9) | 25 (1.5) | 48 (1.7) | 44 (1.5) | 54 (1.8) | 65 (1.8) | 75 (2.2) | 72 (2.1) | 77 (2.0) |
| Missing | 9462 (38.6) | 1149 (70.8) | 1698 (61.2) | 1682 (56.3) | 1303 (43.4) | 1265 (36.6) | 1004 (29.5) | 789 (22.9) | 572 (15.0) |
| **Previous AMI** |  |  |  |  |  |  |  |  |  |
| Yes | 8046 (32.8) | 460 (28.3) | 861 (31.0) | 954 (32.0) | 970 (32.3) | 1174 (34.0) | 1109 (32.6) | 1159 (33.7) | 1359 (35.7) |
| No | 14859 (60.6) | 814 (50.1) | 1499 (54.0) | 1711 (57.3) | 1808 (60.2) | 2156 (62.4) | 2252 (66.1) | 2227 (64.7) | 2392 (62.8) |
| Missing | 1599 (6.5) | 350 (21.6) | 417 (15.0) | 322 (10.8) | 227 (7.5) | 123 (3.6) | 44 (1.3) | 58 (1.7) | 58 (1.5) |
| **Stroke** |  |  |  |  |  |  |  |  |  |
| Yes | 2561 (10.5) | 152 (9.4) | 318 (11.5) | 333 (11.2) | 291 (9.7) | 347 (10.1) | 335 (9.8) | 369 (10.7) | 416 (10.9) |
| No | 19576 (79.9) | 952 (58.6) | 1818 (65.5) | 2185 (73.2) | 2387 (79.4) | 2910 (84.3) | 3005 (88.3) | 2999 (87.1) | 3320 (87.2) |
| Missing | 2367 (9.7) | 520 (32.0) | 641 (23.1) | 469 (15.7) | 327 (10.4) | 196 (5.7) | 65 (1.9) | 76 (2.2) | 73 (1.9) |
| **COPD** |  |  |  |  |  |  |  |  |  |
| Yes | 3759 (15.3) | 242 (14.9) | 476 (17.1) | 482 (16.1) | 462 (15.4) | 554 (16.0) | 494 (14.5) | 499 (14.5) | 550 (14.4) |
| No | 18480 (75.4) | 881 (54.3) | 1662 (59.9) | 2039 (68.3) | 2234 (74.3) | 2742 (79.4) | 2857 (83.9) | 2878 (83.6) | 3187 (83.7) |
| Missing | 2265 (9.2) | 501 (30.9) | 639 (23.0) | 466 (15.6) | 319 (10.3) | 157 (4.6) | 54 (1.6) | 67 (2.0) | 72 (1.9) |
| **In treatment for hypertension** |  |  |  |  |  |  |  |  |  |
| Yes | 8335 (34.0) | 398 (24.5) | 814 (29.3) | 923 (30.9) | 997 (33.2) | 1173 (34.0) | 1245 (36.6) | 1321 (38.4) | 1464 (38.4) |
| No | 14378 (58.7) | 795 (49.0) | 1443 (52.0) | 1676 (56.1) | 1744 (58.0) | 2179 (63.1) | 2145 (63.0) | 2093 (60.8) | 2303 (60.5) |
| Missing | 1791 (7.3) | 431 (26.5) | 520 (18.7) | 388 (13.0) | 264 (8.8) | 101 (2.9) | 15 (0.4) | 30 (0.9) | 42 (1.1) |
| **Diabetes** |  |  |  |  |  |  |  |  |  |
| Yes | 4530 (18.5) | 265 (16.3) | 452 (16.3) | 512 (17.1) | 534 (17.8) | 601 (17.4) | 662 (19.4) | 690 (20.0) | 814 (21.4) |
| No | 18362 (74.9) | 926 (57.0) | 1910 (68.8) | 2146 (71.8) | 2275 (75.7) | 2750 (79.6) | 2708 (79.5) | 2704 (78.5) | 2943 (77.3) |
| Missing | 1612 (6.6) | 433 (26.7) | 415 (14.9) | 329 (11.0) | 197 (6.5) | 102 (3.0) | 35 (1.0) | 50 (1.5) | 52 (1.4) |
| **Amount of alcohol** |  |  |  |  |  |  |  |  |  |
| Maximum14 drinks for women and 21 for men | 16683 (68.1) | 785 (48.3) | 1595 (57.4) | 1945 (65.1) | 2125 (70.7) | 2437 (70.6) | 2418 (71.0) | 2540 (73.8) | 2838 (74.5) |
| More than 14 drinks for women and 21 for men | 1639 (6.7) | 99 (6.1) | 152 (5.5) | 184 (6.2) | 186 (6.2) | 232 (6.7) | 225 (6.6) | 250 (7.3) | 311 (8.2) |
| Missing | 6010 (25.2) | 740 (45.6) | 1030 (37.1) | 858 (28.7) | 695 (23.1) | 784 (22.7) | 762 (22.4) | 654 (19.0) | 660 (17.3) |
| **Smoking** **habits** |  |  |  |  |  |  |  |  |  |
| Smoker | 7101 (29.0) | 499 (30.7) | 842 (30.3) | 909 (30.4) | 881 (29.3) | 1026 (29.7) | 943 (27.7) | 940 (27.3) | 1061 (27.9) |
| No smoker | 17355 (70.8) | 1116 (68.7) | 1932 (69.6) | 2072 (69.4) | 2122 (70.6) | 2419 (70.1) | 2457 (72.2) | 2499 (72.6) | 2738 (71.9) |
| Missing | 48 (0.2) | 9 (0.6) | 3 (0.1) | 6 (0.2) | 2 (0.07) | 8 (0.2) | 5 (0.2) | 5 (0.2) | 10 (0.3) |

*It was not possible to register in and outpatients until 2006

SD: Standard Deviation; LVEF: Left Ventricular Ejection Fraction; NYHA: New York Heart Association-classification; AMI: Acute Myocardial Infarction; COPD: Chronic Obstructive Pulmonary Disease.
